# Supplementary material for: The extended recovery ring-stage survival assay provides a superior association with patient clearance half-life and increases throughput
Source: Malar J. 2020 Jan 31;19:54. doi: 10.1186/s12936-020-3139-6 (PMC6995136; doi:10.1186/s12936-020-3139-6)
Supplement: Supplementary file 1 — Additional file 1. Table of primer and cycling conditions used for quantitative PCR. The forward and reverse primer sequences of the pfcrt gene used in this study and the cycling conditions used in the fast mode of the ABI 7900HT. Primer sequence was created using Integrated DNA Technologies’ (IDT) qPCR primer design software. Using fold change as the readout for qPCR (comparing the treated sample to the untreated sample of the same parasite isolate) avoids any amplification bias between different parasite isolates. [file 12936_2020_3139_MOESM1_ESM.pdf]

	Sequence
Forward	GGGTGATGTTGTAAGAGAACCA
Reverse	ACGAACAAGCCATTTGATATTACAC

Cycling conditions:
95º C 20 seconds
95º C 1 second
62.3 º C 30 seconds
65º C 15 seconds
