# Supplementary figures and images for: The extended recovery ring-stage survival assay provides a superior association with patient clearance half-life and increases throughput
Source: Malar J. 2020 Jan 31;19:54. doi: 10.1186/s12936-020-3139-6 (PMC6995136; doi:10.1186/s12936-020-3139-6)

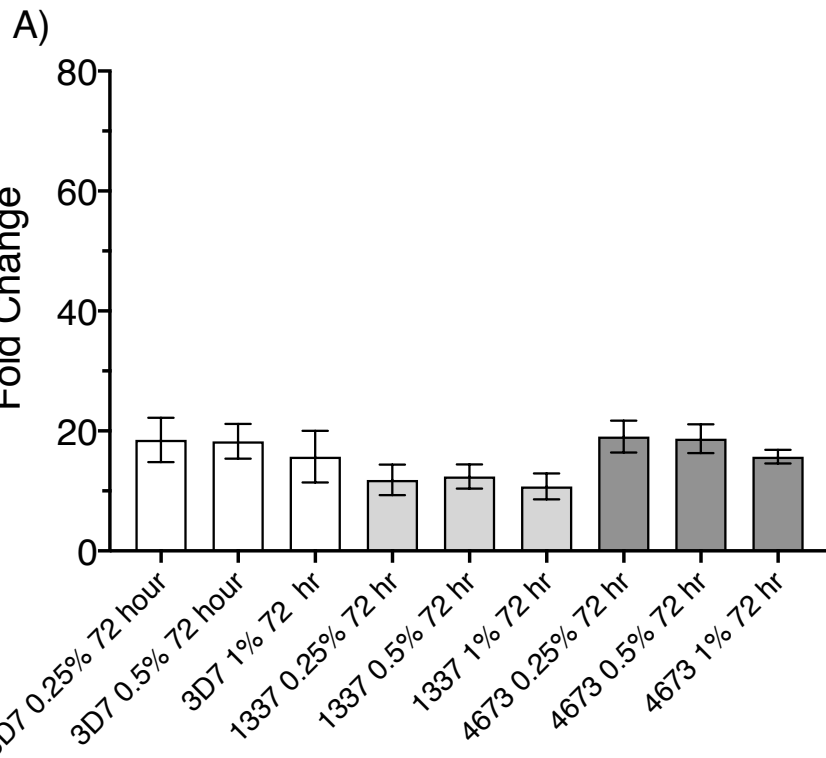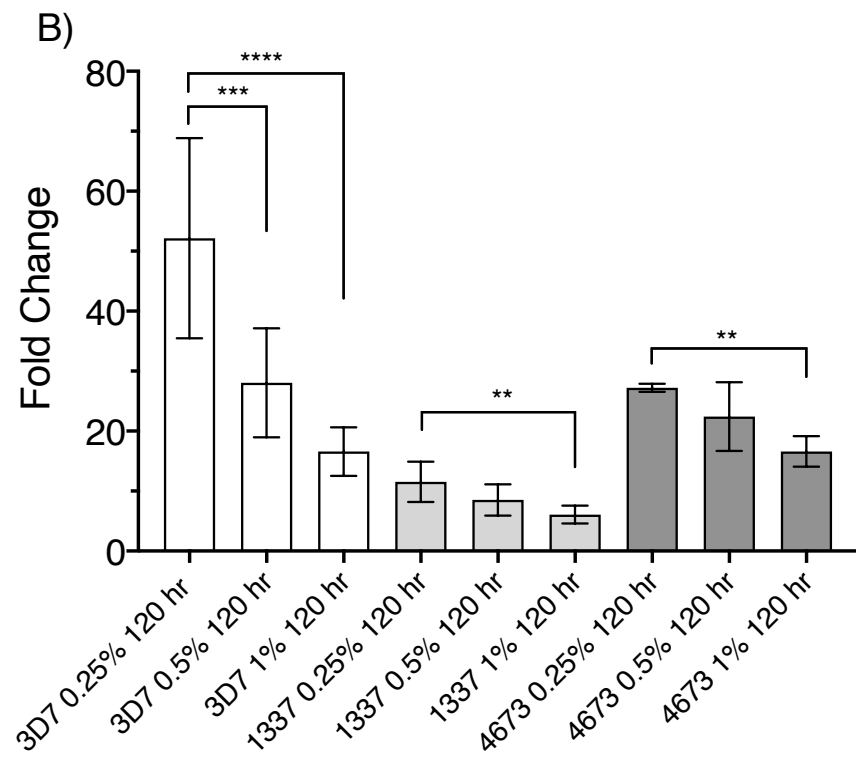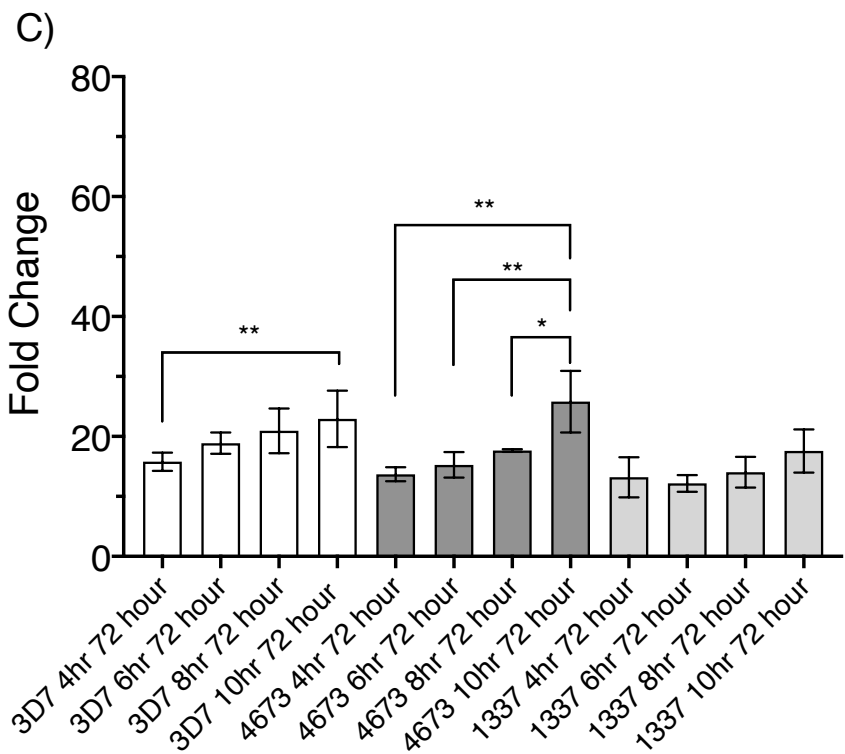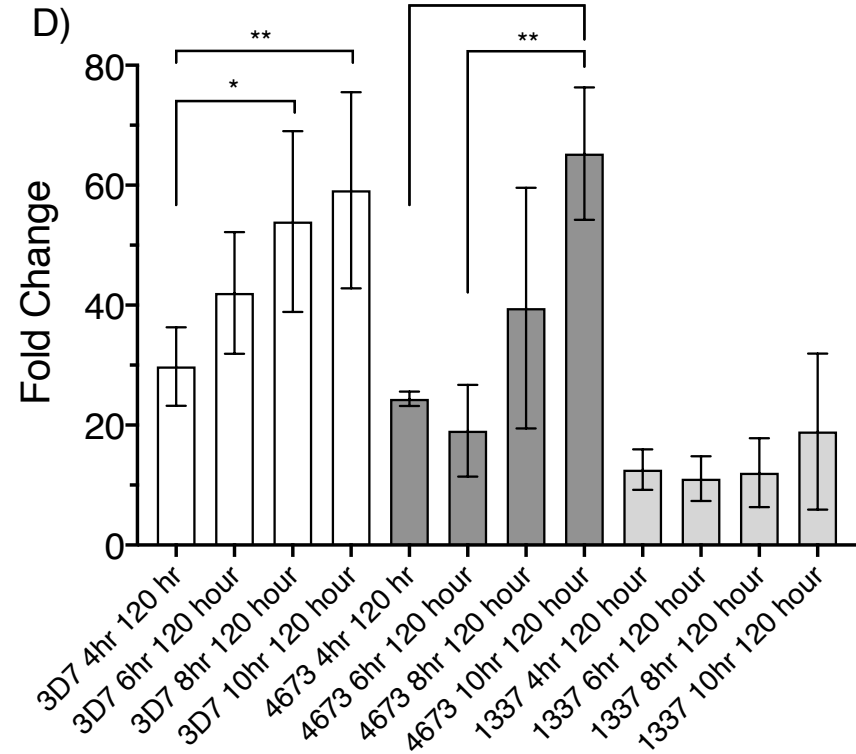

Supplement: Supplementary file 2 — Additional file 2. Effects of assay modification on final assay readout. (A) The starting parasitaemia for the assay was set at 0.25%, 0.5%, or 1% (as measured by microscopy) for three different parasite isolates (3D7, 1337, and 4673) and the fold change between the treated (DHA) and untreated (DMSO) samples was measured with three biological replicates (each with three technical replicates) at 72 h post-drug treatment and (B) at 120 h post-drug treatment. After determining that a 0.25% parasitaemia measured by microscopy was equivalent to 0.5% parasitaemia measured by flow cytometry (C), the starting parasitaemia was determined using flow cytometry and set to 0.5% and the time from Percoll synchronization to drug (DHA or DMSO) application was varied (4 h, 6 h 8 h, or 10 h) for the three different parasite isolates (3D7, 1337, and 4673) and the fold change between treated and untreated samples was measured with three biological replicates (each with three technical replicates) at 72 h post-drug treatment and (D) 120 h post-drug treatment. The effect of varying starting parasitaemia and time to drug treatment post-synchronization on fold change was calculated using a one-way ANOVA. Statistically significant differences between treatments are reported on the corresponding figures. [file 12936_2020_3139_MOESM2_ESM.pdf]

Fold Change

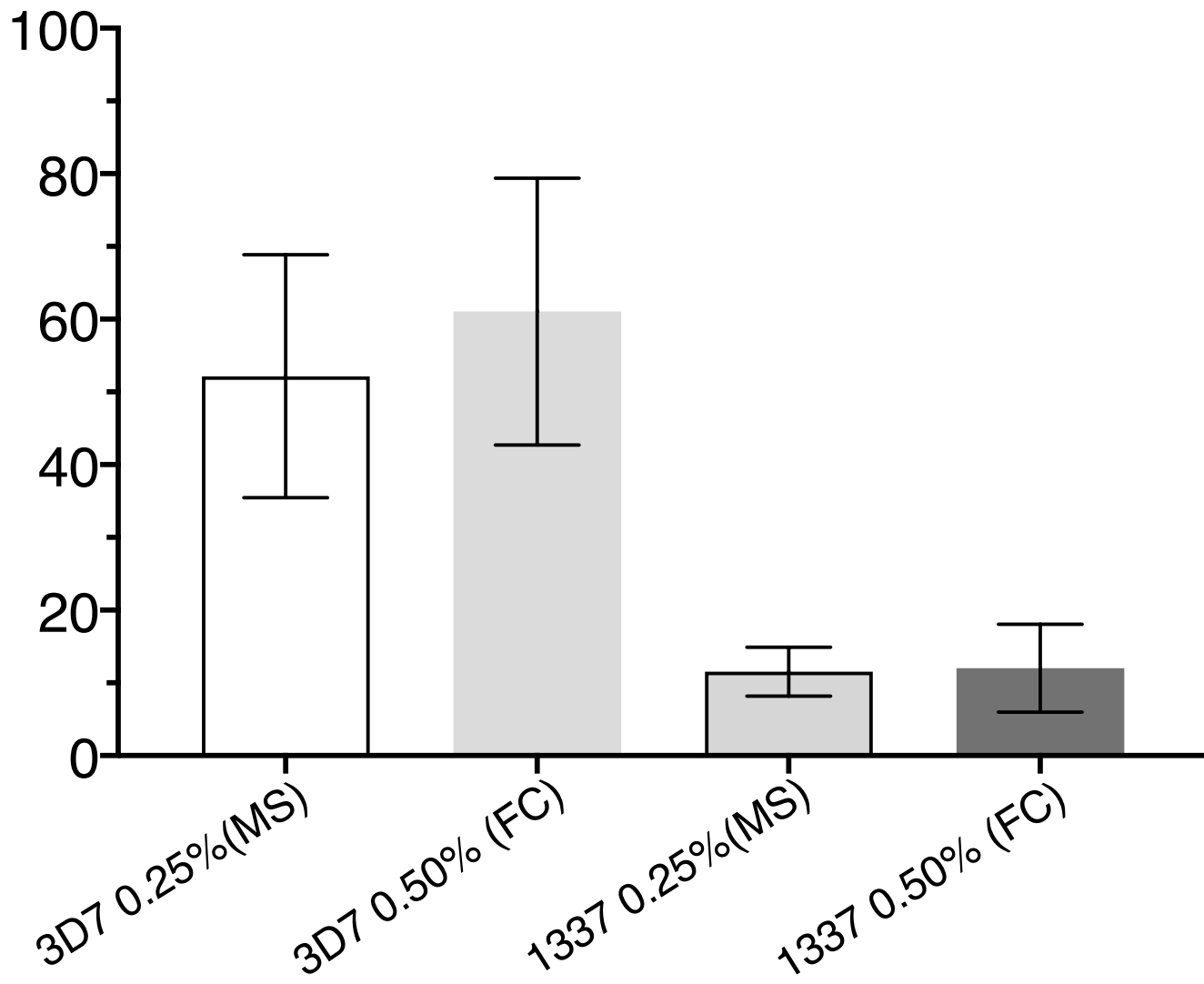

Supplement: Supplementary file 3 — Additional file 3. Comparison of microscopy vs flow cytometry to calculate starting parasitaemia. Fold change data for two parasite lines (3D7 and 1337) used as controls in eRRSA show no significant difference (p = 0.21 and 0.20, respectively) between a starting parasitaemia of 0.25% by microscopy (MS) and a starting parasitaemia of 0.5% determined by flow cytometry (FC). P-values were calculated using an unpaired t-test with Welch’s correction. [file 12936_2020_3139_MOESM3_ESM.pdf]

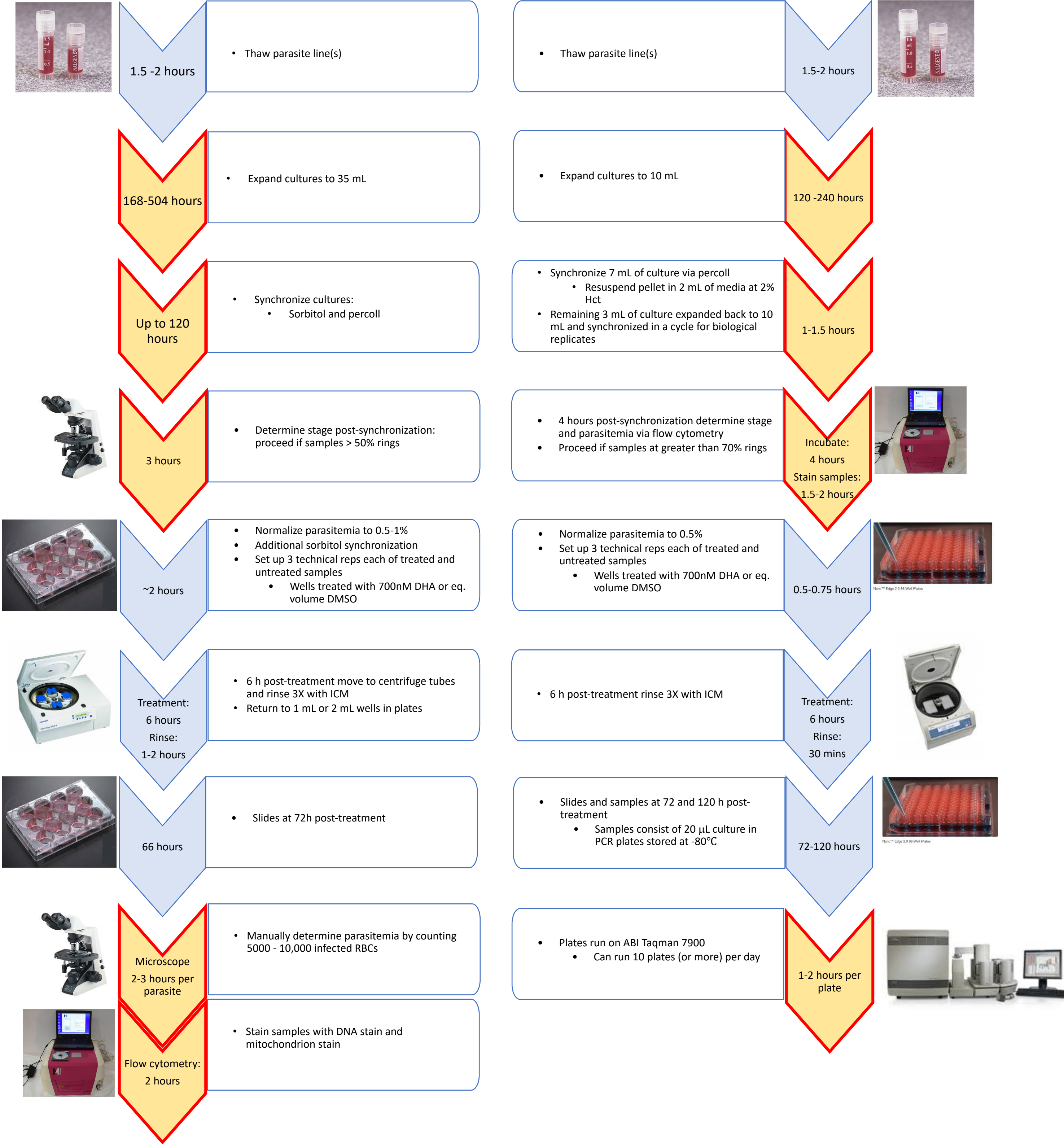

Supplement: Supplementary file 4 — Additional file 4. Summary of modifications for the eRRSA compared to the standard RSA. A timeline comparing the various steps and the estimated time needed for each step of the standard WWARN RSA [14] (left) and the eRRSA (right). There are limiting steps in both the eRRSA and RSA (marked with orange arrows), but the eRRSA allows for more parasite isolates with more biological replicates to be done in less time compared to the standard RSA. [file 12936_2020_3139_MOESM4_ESM.pdf]
