# Supplementary material for: The extended recovery ring-stage survival assay provides a superior association with patient clearance half-life and increases throughput
Source: Malar J. 2020 Jan 31;19:54. doi: 10.1186/s12936-020-3139-6 (PMC6995136; doi:10.1186/s12936-020-3139-6)
Supplement: Supplementary file 5 — Additional file 5. Comparison of previously published correlation rates of RSA results with PC1/2. Previous publications where PC1/2 and RSA results on the same samples were reported are listed here. If RSA results were available, then the Spearman correlation between PC1/2 and RSA was calculated. If the Spearman correlation was not provided, it was internally calculated and is marked with an*. [file 12936_2020_3139_MOESM5_ESM.pdf]

First Author	Year	Number of Isolates	RSA and PC1/2 Correlations	Journal	doi
Witkowski	2013	30	0.5476	Lancet Infect Dis.	10.1016/S1473-3099(13)70252-4
Amaratunga	2014	44	0.48511225	Antimicrobial Agents and Chemotherapy	10.1128/AAC.03055-14
Ariey	2014	49	NA	Nature	10.1038/nature12876
Wang	2015	182	NA	Antimicrobial Agents and Chemotherapy	10.1128/AAC.01255-15
Ye	2016	111	NA	Scientific Reports	10.1038/srep20100
Boullé	2016	25	NA	Emerg Infect Dis.	10.3201/eid2208.160004
Menard	2016	64	NA	Malaria Journal	10.1186/s12936-016-1622-x
Thanh	2017	9	NA	Malaria Journal	10.1186/s12936-017-1680-8
Mukherjee	2017	36	0.3534*	Malaria Journal	https://doi.org/10.1186/s12936-017-1845-5
Sá	2018	22	NA	PNAS	10.1073/pnas.1813386115
Das	2019	226	NA	Clinical Infectious Diseases	10.1093/cid/ciy1038
Phong	2019	25	NA	Malaria Journal	10.1186/s12936-019-2640-2
